# Characterization of the T Cell Response to *Lactobacillus casei* Cell Wall Extract in Children With Kawasaki Disease and Its Potential Role in Vascular Inflammation

**DOI:** 10.3389/fped.2021.633244

**Published:** 2021-02-19

**Authors:** Li-En Hsieh, Adriana H. Tremoulet, Jane C. Burns, Magali Noval Rivas, Moshe Arditi, Alessandra Franco

**Affiliations:** ^1^Department of Pediatrics, School of Medicine, University of California San Diego, La Jolla, La Jolla, CA, United States; ^2^Division of Infectious Diseases and Immunology, Departments of Pediatrics, Infectious and Immunologic Diseases Research Center (IIDRC), Los Angeles, CA, United States; ^3^Department of Biomedical Sciences, Cedars-Sinai Medical Center, Los Angeles, CA, United States; ^4^Department of Pediatrics, David Geffen School of Medicine at University of California Los Angeles, Los Angeles, CA, United States

**Keywords:** Kawasaki disease, *Lactobacillus casei* cell wall extract, T cells, T cell homing, human T cells

## Abstract

KD is an acute febrile illness and systemic vasculitis of unknown etiology among young children, which can cause coronary artery abnormalities and aneurysms (CAA) and is the leading cause of acquired heart disease among children in the US. *Lactobacillus casei* cell wall extract (LCWE) induces in mice a vasculitis following intraperitoneal injection defined by the activation of macrophages, dendritic cells and CD8+ cytotoxic T cells leading to aortitis, coronary arteritis, aneurysms and myocarditis that strongly mimic the immunopathology and the cardiac lesions observed in children with Kawasaki disease (KD). To address a potential pathogenic role of LCWE-specific T cells in human vascular inflammation, we studied the activation of circulating CD4+ and CD8+ T cells *ex vivo* in response to LCWE in 3 cohorts: (1) KD children 2–3 weeks after fever onset, (2) age-similar healthy children controls, (3) healthy adult controls. In all subjects studied, pro-inflammatory CD4+ and CD8+T cells responded to LCWE with no significant differences. Peripherally-induced regulatory T cells (iTreg) also responded to LCWE and potentially reverted to Th17, as suggested by the detection of IL-17 in culture supernatants. Central memory T cells were also detectable and were more abundant in adults. The potential homing to the vessels of LCWE-specific T cells was suggested by the expression of CCR6 and CD31. In conclusion, a non-pathogenic, LCWE-specific T cell repertoire could lead to KD depending upon priming conditions, genetic factors and immune activation by other antigens.

## Introduction

*Lactobacillus casei* is a probiotic commensal bacterium that is naturally found in the gut and has an important role in maintaining mucosal homeostasis by activating T cells that “sense” other pathogens and serve as a by-stander source of lymphokines and co-stimulatory signals (1). In fact, in many therapeutic settings including neoplasms, *L. case*i serves as an adjuvant to facilitate the activation of tumor-specific T cells (2, 3). However, an intraperitoneal injection of its cell wall extract, *L. casei* cell wall extract (LCWE), induces vasculitis in mice and is a well-recognized murine model to study KD, an acute pediatric vasculitis of the coronary arteries that affects infants and young children (4–6). The cascade of events in the LCWE-induced KD murine model include abnormalities of mucosal permeability and intestinal leakage and the subsequent release of pro-inflammatory cytokines such as IL-1β, IL-6, and TNF that recruit monocytes, macrophages, and T cells to the inflamed vascular tissues (5, 7).

We explored the possibility that LCWE may cross-react with human immune cells, particularly T cells, and may participate in KD development and pathogenesis. Therefore, we characterized *in vitro* the pro-inflammatory CD8+ and CD4+ T and regulatory T (Treg) cell responses to LCWE in peripheral blood mononuclear cells (PBMC) isolated from subacute KD children, healthy children with a history of KD, and healthy adults.

The study suggests that LCWE is immunogenic for pro-inflammatory T helper (Th) 1, Th17, and CD8+ T cells and inducible regulatory T cells (iTreg) in all 3 cohorts studied. Chemokine receptor expression and markers that indicated homing to the vascular compartment were expressed in 20–30% of the T cells activated by LCWE with potential implications for participating in vascular inflammation.

## Materials and Methods

### Study Population

The study protocols for KD subjects, healthy children, and adult subjects were approved by the Institutional Review Board at the University of California San Diego (IRB #140220 and #101213, respectively). The pediatric subjects were enrolled at Rady Children's Hospital, San Diego, following written parental informed consent and patient assent as appropriate. The adult subjects were enrolled from the Normal Blood Donor services at The Scripps Research Institute, San Diego following written consent.

Ten subacute, IVIG-treated KD subjects 2 to 4 weeks after disease onset, 9 healthy children who recovered from KD at least 1 year previously, and 10 healthy adult donors were enrolled in the study (Table 1).

**Table 1:** Subjects enrolled in this study.

**KD subjects**						**Healthy children with a history of KD**						**Healthy adults**			
**KD subject**	**Days after onset**	**Age (yrs)**	**Sex**	**Ethnicity**	**zMAX score**	**HC subject**	**Years after onset**	**Age (yrs)**	**Sex**	**Ethnicity**	**zMAX score**	**ND subject**	**Age (yrs)**	**Sex**	**Ethnicity**
1	25	0.4	M	Hispanic	1.58	1	6	7	F	Caucasian	1	1	40	F	Caucasian
2	24	7.9	F	Asian	1.11	2	1	3	M	Hispanic	1.85	2	29	M	Asian
3	20	5.9	F	Mixed	0.44	3	3	4	M	Asian	3.12	3	40	F	Mixed
4	14	1.9	M	Asian	0.94	4	1	2	M	Hispanic	3.4	4	28	M	Mixed
5	27	0.6	M	Hispanic	2.52	5	6.5	9	F	Hispanic	0.89	5	51	M	Hispanic
6	19	9.5	M	Brazilian	3.24	6	2.5	4	F	Mixed	1.42	6	25	M	Asian
7	18	6.1	M	Caucasian	0.94	7	2	3	F	Caucasian	1.02	7	45	F	Mixed
8	23	3.5	M	Caucasian	1.32	8	1	5	M	Hispanic	0.61	8	64	F	Non-Hispanic
9	21	7.8	M	Hispanic	0.5	9	1	7	M	Mixed	1.76	9	59	F	Caucasian
10	20	3.5	F	Mixed	1.1							10	33	F	Asian

### LCWE Preparation

LCWE was prepared as previously described (5, 8). In brief, *Lactobacillus casei* (ATCC 11578) were grown in Lactobacillus MRS broth (Difco) for 48 h, collected, and washed with 1X PBS. The bacteria were disrupted using 4% SDS/PBS for 18 h at the room temperature. Cell wall fragments were washed 8 times with 1X PBS to remove the SDS followed by sonication for 2 h with a 3/4-inch horn and a garnet tip at maximum power. The cell wall fragments were kept in a dry ice/ethanol bath during the sonication. The cell wall fragments were centrifuged for 20 min at 12,000 rpm, 4°C. The supernatants were collected, centrifuged for 1 h at 38,000 rpm, 4°C, and the pellet was discarded. The total rhamnose content of the cell wall extract was determined by a colorimetric phenol-sulfuric assay. The LCWE preparation was LPS free. The extract is obtain directly using the bacterial and sonicating it to obtain cell wall components. *L. Casei* is a Gram positive bacteria therefore do not contain LPS. In addition, Dr. Arditis' laboratory has established that LCWE *in vitro* and *in vivo* models utilizes TLR2 dependent and not TLR4 as LPS. LCWE in a PAGE gel with LPS control then stained with Pro-Q Emerald 300 polysaccharide staining kit did not show LPS like bands (Supplementary Figure 1). Endotoxin levels were measured below 0.5 EU/mg, therefore negative.

### T Cell Culture Conditions and Characterization of T Cell Responses to LCWE

Peripheral blood mononuclear cells (PBMC) were separated from heparinized whole blood by Ficoll Histopaque density gradient (Sigma). 4 × 10^5^ PBMC/well were stimulated with 1 and 10 μg/ml of LCWE in 96 well-flat-bottom plates (Falcon) in the absence of exogenous IL-2 for 4 days. T cell responses to LCWE were assessed by 2 different methods. First, we used flow cytometry to enumerate DR+ activated CD4+ and CD8+ T cells in combination with markers to address their homing (CCR6 and CCR7), memory status (IL-15R), and the expression of CD31. CD4+ CD25^high^ Treg were further defined by the expression of the IL-7R that is uniquely expressed on iTreg but not on thymic-derived natural Treg (nTreg). Second, we measured IL-2, IFNγ, IL-10, and IL-17 in culture supernatants by ELISA. Agonistic anti-CD3 (clone HIT3a, mouse IgG2aκ, Biolegend) and agonistic anti-CD28 (clone CD28.2, mouse IgG1κ, BD Bioscience) were used as controls for the T cell activation.

To enumerate recently activated CD4+ and CD8+ T cells, we used in combination anti-human CD4 PerCp/Cy5.5 (clone RPA-T4, mouse IgG1κ, eBioscience), anti-human CD8 AF700 (mouse IgG1κ, clone RPA-T8, BD Bioscience), and anti-human HLA-DR (APC/H7, clone G46-6, mouse IgG2aκ (BD Bioscience). To characterize central and effector memory T cells (Tcm and Tem), the surface expression of IL-15R and CCR7 on CD4+ and CD8+ T cells was established by anti-human IL-15R (clone eBioJM7A4, mouse IgG2bκ) and anti-human CCR7 PE (clone 3D12, mouse IgG2aκ) from eBioscience. To address the homing of CD4+ and CD8+ T cells to the vessels and the gut, the surface expression of CCR6 was evaluated on LCWE-specific T cells by anti-human CCR6 APC (clone R6H1, mouse IgG1κ, eBioscience). The expression of CD31 was evaluated on LCWE-specific CD4+ and CD8+ T cells with anti-human CD31 BV421 (clone WM-59, mouse IgG1κ, BD Bioscience). Data were acquired on BD FACSCanto II (BD Bioscience) and analyzed with FlowJo software version 10 (Tree Star). IL-2 and IFNγ in culture supernatants were measured by coating plates with 2 μg/ml of anti-human IL-2 and anti-human IFNγ primary antibodies and 2 μg/ml of biotin labeled secondary antibodies (BD Bioscience). IL-10 in culture supernatants was measured by coating 4 μg/ml of anti-human IL-10 primary antibody and 2 μg/ml of biotin labeled secondary antibody (BD Bioscience). IL-17 in culture supernatants was measured by coating 1 μg/ml of primary antibody and 1 μg/ml of secondary antibody (Invitrogen).

## Results

### Pro-Inflammatory T Helper 1 CD4+ T Cells and CD8+ Cytotoxic T Cells Responded to LCWE *in vitro*

To explore a possible role for LCWE-specific T helper 1 (Th1) and CD8+ cytotoxic (CTL) T cell responses in the pathogenesis of KD, we enrolled 3 different cohorts: (1) subacute KD children 2–4 weeks after disease onset (*n* = 7), (2) healthy children with a previous history of KD at least 1 year previously (*n* = 9), and (3) healthy adults (*n* = 7) (Table 1). PBMC from individual subjects were stimulated with a scalar dose (1 and 10 μg/ml) of LCWE for 4 days in the absence of exogenous interleukin (IL)-2. Th1 and cytotoxic T lymphocyte (CTL) responses were tested by (1) enumeration of CD4+ HLA-DR+ and CD8+ HLA-DR+ activated T cells by flow cytometry and (2) measurement of IL-2 and IFNγ secretion in the culture supernatants.

**Figure 1:**
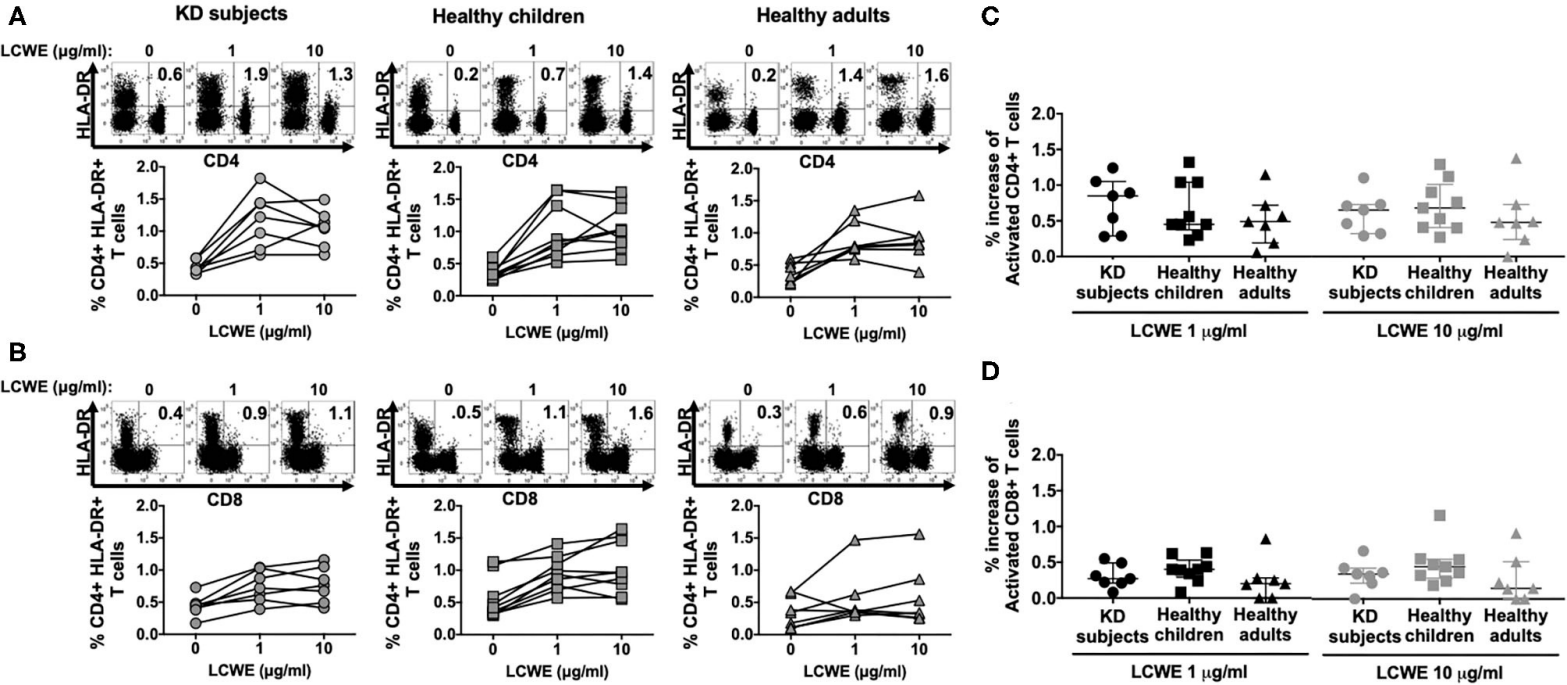
Activation of circulating CD4+ and CD8+ T cells in response to LCWE. PBMC from KD subjects (*n* = 7) 2 to 4 weeks after disease onset, healthy children (*n* = 9), and healthy adults (*n* = 7) were stimulated with 1 and 10 μg/ml of LCWE for 4 days and the percent activated CD4+ HLA-DR+ **(A)** and activated CD8+ HLA-DR+ **(B)** T cells were enumerated by flow cytometry with specific monoclonal antibodies. Unstimulated PBMC cultures served as controls in these experiments. A representative FACS plot of HLA-DR expression on CD4+ and CD8+ T cells for each cohort is shown. Percent increase of CD4+ HLA-DR+ **(C)** and CD8+ HLA-DR+ **(D)** T cells in response to 1 μg/ml (dark symbols) and 10 μg/ml (light symbols) of LCWE vs. un-stimulated control. The data are shown with median and interquartile range. Each symbol is the result derived from each subject tested. The statistical differences between cohorts were tested by one-way ANOVA. A *P* < 0.05 was considered significant. The results suggested that LCWE stimulates CD4+ (1 μg/ml, *p* = 0.5564; 10 μg/ml, *p* = 0.6553) and CD8+ (1 μg/ml, *p* = 0.3947; 10 μg/ml, *p* = 0.3405) T cells in the 3 cohorts with no statistical differences.

In all the KD subjects studied, LCWE stimulated both CD4+ and CD8+ T cells as shown by the expression of the activation marker DR (Figures 1A,B). CD4+ and CD8+ T cells from healthy children and healthy adults showed a similar magnitude of activation following LCWE stimulation (Figure 1). Agonistic stimulation with anti-CD3 and anti-CD28 served as controls for T cell activation (Supplementary Figure 2).

In KD subjects, the median percent increase of CD4+ HLA-DR+ T cells in PBMC stimulated with 1 μg/ml and 10 μg/ml of LCWE was 0.85% (IQR: 0.42–0.97%) and 0.65% (IQR: 0.39–0.72%), respectively (Figure 1C). In healthy children, the median percent increase of CD4+ HLA-DR+ T cells in PBMC stimulated with 1 and 10 μg/ml of LCWE was 0.45% (IQR: 0.45–1.04%) and 0.68% (IQR: 0.41–0.90%), respectively (Figure 1C). In healthy adults, the median percent increase of CD4+ HLA-DR+ T cells in PBMC stimulated with 1 and 10 μg/ml of LCWE extract was 0.49% (IQR: 0.32–0.56%) and 0.48% (IQR: 0.36–0.61%), respectively (Figure 1C). Similar results were obtained when we looked at the expansion of CD8+ cytotoxic T cells (CTL) in response to the LCWE extract. In KD subjects, the median percent CD8+ HLA-DR+ T cells increase in PBMC stimulated with 1 and 10 μg/ml of LCWE extract was 0.27% (IQR: 0.22–0.40%) and 0.35% (IQR: 0.27–0.40%), 0.40% (IQR: 0.34–0.44%), and 0.45% (IQR: 0.33–0.50%) in healthy children 0.20% (IQR: 0.10–0.27%) and 0.15% (IQR: 0.07–0.38%) in healthy adults (Figure 1D).

**Figure 2:**
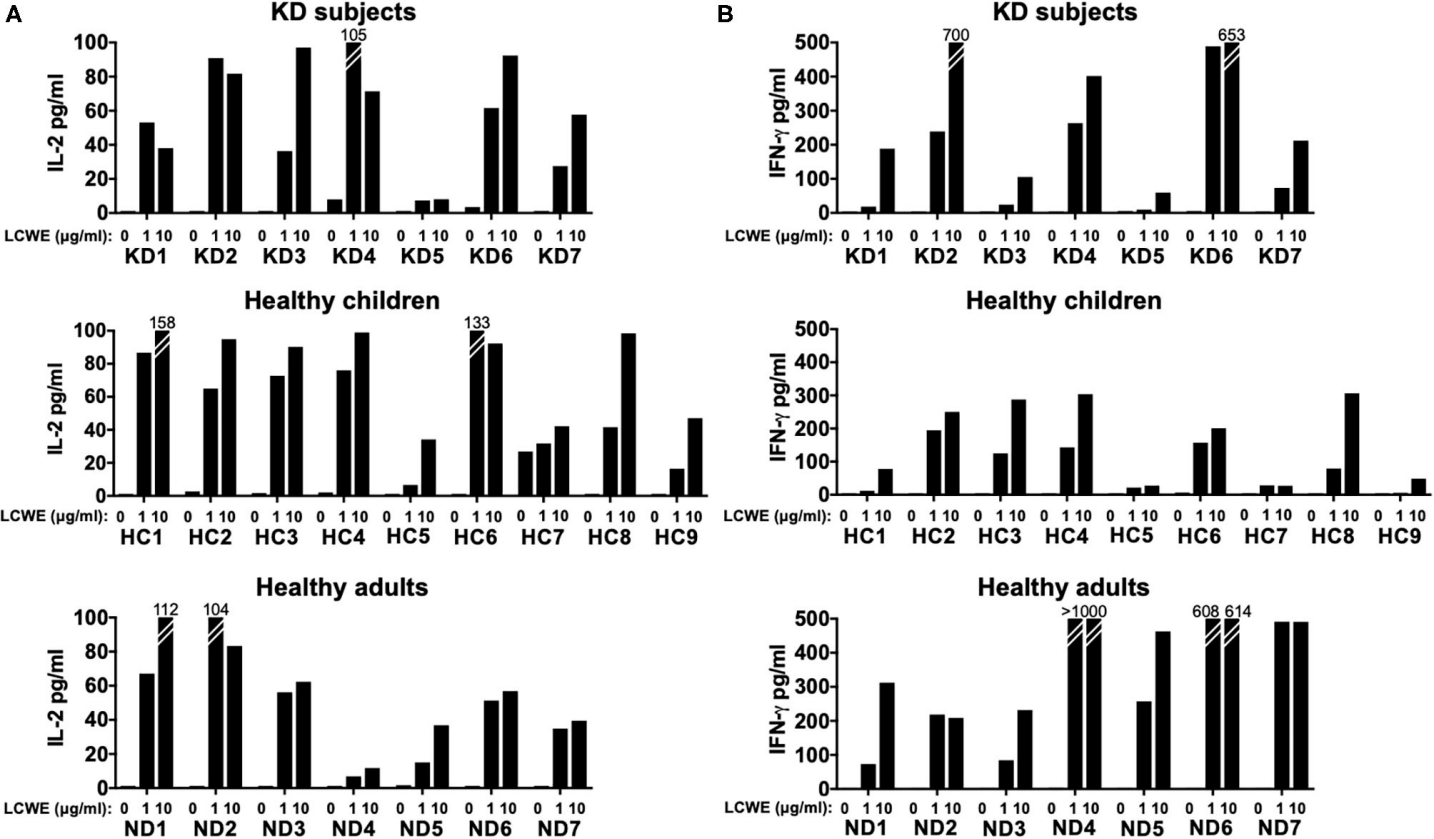
IL-2 and IFNγ production by T cells in response to LCWE. To validate the antigen-specific response to LCWE, we measured IL-2 **(A)** and IFNγ **(B)** secretion in culture supernatants. CD4+ and CD8+ T cells secreted IFNγ, suggesting a pro-inflammatory T helper 1 and cytotoxic functional phenotype in response to LCWE.

T cell activation was supported by the measurement of IL-2 secretion in the culture supernatants (Figure 2A). IFNγ was also measurable in a dose-dependent manner in PBMC cultures from the 3 cohorts stimulated with LCWE extract (Figure 2B). The results suggested that the LCWE extract was highly immunogenic and stimulated Th1 and CTL *in vitro* not only in subacute KD children, but also in healthy children with a previous history of KD and adult controls. Th17 were also capable of secreting IFNγ and their role in this antigenic model was further explored.

### iTreg Expand in Response to LCWE and Potentially Revert to Th17

To address whether LCWE extract also stimulates Treg, we enrolled 3 additional sub-acute KD subjects (KD8–10), 5 healthy children (HC5–9), and 3 additional healthy adults (ND8–10) (Table 1).

**Figure 3:**
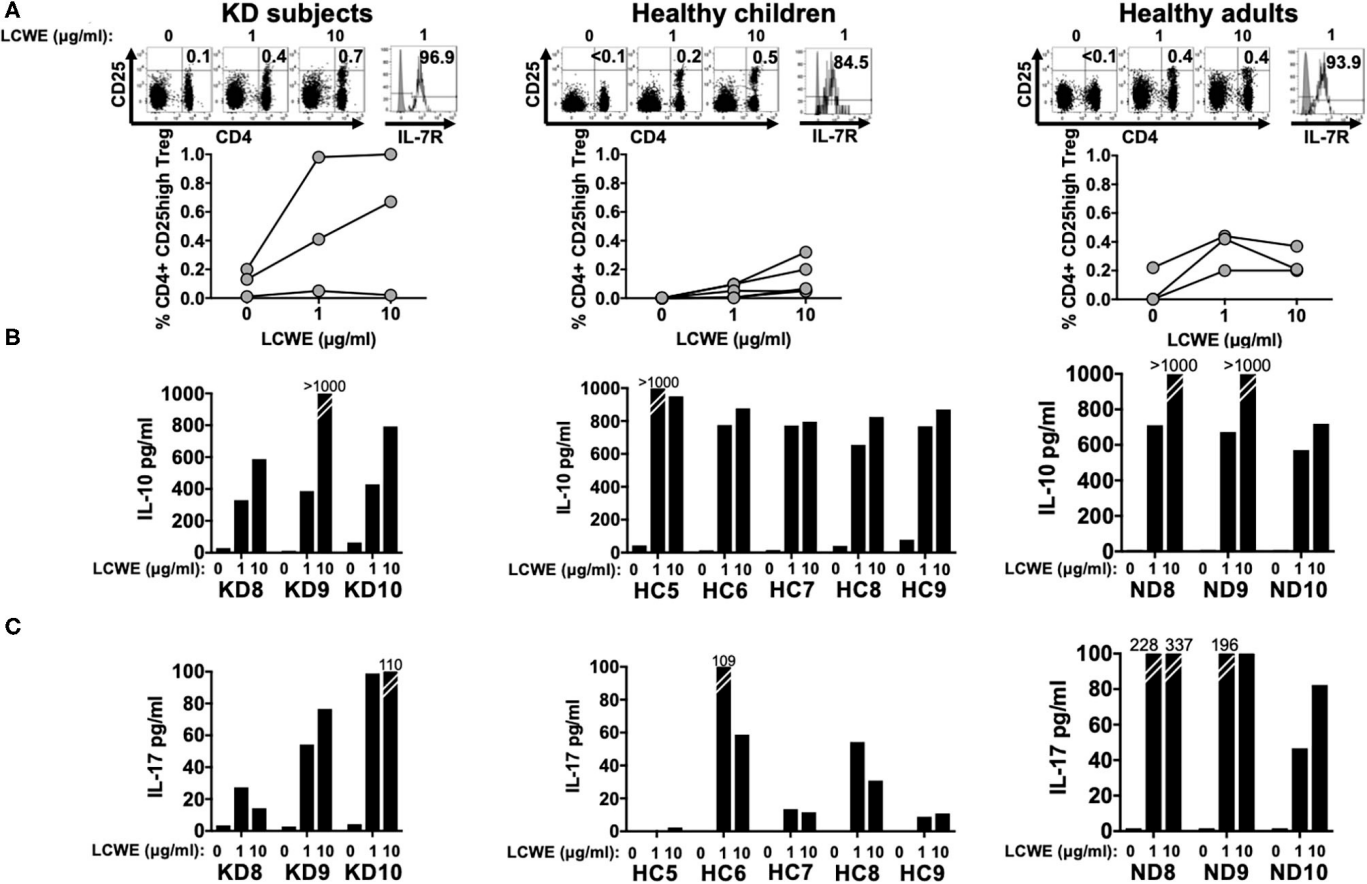
CD4+ CD25^high^ Treg expansion in response to LCWE. To address peripherally-induced Treg responses to LCWE, we studied CD4+ CD25^high^ Treg expansion in PBMC stimulated with 1 and 10 μg/ml of LCWE for 4 days in 3 subacute KD subjects 3 weeks after disease onset (KD8–10, Table 1), 5 healthy children (HC5–9, Table 1), and 3 healthy adults (ND8–10, Table 1). Unstimulated PBMC served as control **(A)** Representative CD4+ CD25^high^ T cell enumeration and IL-7R expression is shown. Gray: un-stained control. Culture supernatants derived from the same PBMC cultures were tested for IL-10 **(B)** and IL-17 **(C)** secretion by ELISA. The statistical differences of the Treg responses between cohorts were tested by one-way ANOVA. A *P* < 0.05 was considered significant. The results suggested that LCWE stimulates Treg (1 μg/ml, *p* = 0.0974; 10 μg/ml, *p* = 0.1960) in the 3 cohorts with no statistical differences.

After 4 days in culture, PBMC stimulated with LCWE extract were collected and Treg cell expansion was assessed by flow cytometry by enumerating CD4+ CD25^high^ T cells. All subjects studied showed CD4+ CD25^high^ T cell expansion in response to LCWE extract and the CD4+ CD25^high^ T cells expressed high levels of IL-7R, suggesting that CD4+ CD25^high^ T cells were peripherally induced Treg (iTreg) and not natural, thymic-derived Treg (nTreg). iTreg arise from pro-inflammatory Th17 cells under repeated stimulation and convert from Th17 depending upon RoRγt expression (9–11). The median percent increase of CD4+ CD25^high^ Treg in PBMC stimulated with 1 μg/ml of LCWE was 0.28% (IQR: 0.16–0.53%) in subacute KD subjects, 0.05% (IQR: 0.01–0.01%) in healthy children, and 0.22% (IQR: 0.21–0.32%) in healthy adults (Figure 3A). The median percent increase of CD4+ CD25^high^ Treg in PBMC stimulated with 10 μg/ml of LCWE was 0.54% (IQR: 0.28–0.67%) in subacute KD subjects, 0.06% (IQR: 0.05–0.2%) in healthy children, and 0.20% (IQR: 0.17–0.20%) in healthy adults (Figure 3A). IL-10 secretion in the culture supernatants was consistent with the expansion of iTreg following LCWE extract stimulation with a dose-dependent IL-10 response (Figure 3B). As previously observed for the pro-inflammatory Th1 and CTL responses, the iTreg expansion in response to LCWE extract showed no differences among the 3 cohorts. We were also able to detect IL-17 secretion by T cells in all of the subjects except for one of the healthy children (HC5) (Figure 3C).

### T Cell Memory to LCWE

Next, we addressed the central and effector memory phenotypes of LCWE-specific T cells in 3 subacute KD subjects (KD5 – 7), 5 healthy children (HC5 – 9), and 2 healthy adults (ND3–4) (Table 1). Memory T cells were defined by the expression of IL-15R on activated CD4+ and CD8+ T cells. Central and effector memory T cells were defined by IL-15R+ T cells with or without co-expressing the chemokine receptor for homing to the lymph nodes, CCR7, respectively (12).

**Figure 4:**
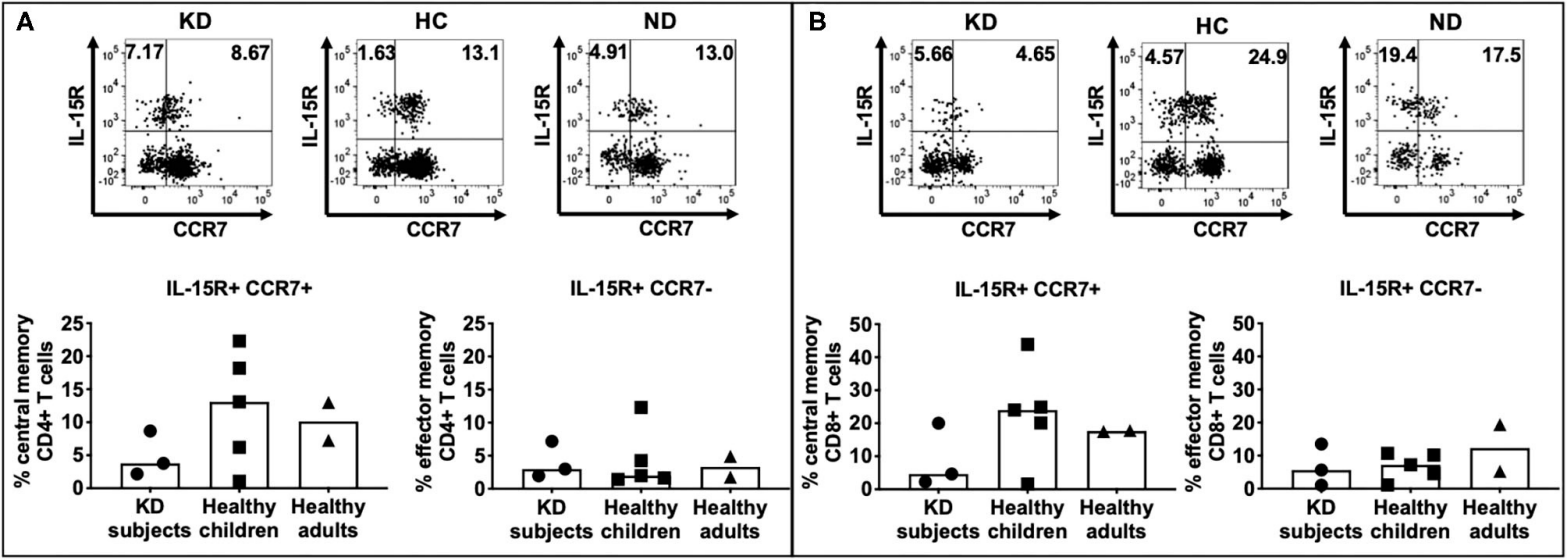
Enumeration of effector and central memory LCWE-specific CD4+ and CD8+ T cells. PBMC from subacute KD subjects (*n* = 3; KD 5–7, Table 1), healthy children (*n* = 5; HC 5–9, Table 1), and healthy adults (*n* = 2; ND3–4, Table 1) were stimulated with LCWE (1 μg/ml) for 4 days and studied for the expression of IL-15R and CCR7+ on CD4+ HLA-DR+ **(A)** and CD8+ HLA-DR+ **(B)** T cells were studied by flow cytometry to enumerate central memory (IL15R+ CCR7+) and effector memory (IL-15+ CCR7-) T cells. A representative FACS plot of IL-15R+ CCR7+ central memory T cells and IL-15R+ CCR7- effector memory T cells from each cohort is shown. In all 3 cohorts, both CD4+ and CD8+ memory T cells were detectable suggesting previous and repeated exposure to the antigen. As expected, effector memory T cells were less numerous than central memory T cells (1.43–22.4% vs. 1.08–12.3% CD4+ T cells and 1.59–43.9% vs. 1.06–19.4% CD8+ T cells, respectively). The data are shown with median and interquartile range. Each symbol is the result derived from each subject tested. The statistical differences between cohorts were tested by one-way ANOVA. A *P* < 0.05 was considered significant. No difference of CD4+ (central memory, *p* = 0.4014; effector memory, *p* = 0.9547) and CD8+ (central memory, *p* = 0.3662; effector memory, *p* = 0.5224) central memory and effector memory T cells were found among the 3 cohorts.

Both LCWE specific CD4+ (Figure 4A) and CD8+ (Figure 4B) memory T cells were identified in all 3 cohorts (Supplementary Figure 3). CD4+ central memory T cells were more prevalent than CD4+ effector memory T cells in subacute KD subjects (2.17–8.67% vs. 1.96–7.17%), healthy children (1.08–22.3% vs. 1.43–12.3%), and healthy adults (7.26–13% vs. 1.75–4.91%). Similarly, CD8+ central memory T cells were more abundant than CD8+ effector memory cells in the 3 cohorts: 2.27–20% vs. 1.06–13.5% in subacute KD children; 1.59–43.9% vs. 1.14–10.2% in healthy children; 17.5–17.8% vs. 5.25–19.4% in healthy adults. There was no difference in the percent of central and effector memory CD4+ and CD8+ T cells among the 3 cohorts. Altogether, the presence of memory T cells suggested a previous exposure to the antigen that leads to central memory CD4+ and CD8+ T cells.

### Homing to the Vessels of LCWE-Specific T Cells

To understand the potential role of LCWE-specific T cells in the pathogenesis of vascular inflammation, we next studied the expression of CCR6, a chemokine receptor that contributes to atherogenesis and vascular homing, and CD31, an adhesion molecule that is important for the trans-endothelial migration of T cells and is a regulator of T cell activation. The expression of CCR6 and CD31 on T cells after LWCE stimulation was studied in 3 subacute KD subjects (KD5–7), 5 healthy children (HC5–9), and 2 healthy adults (ND3–4) (Table 1).

**Figure 5:**
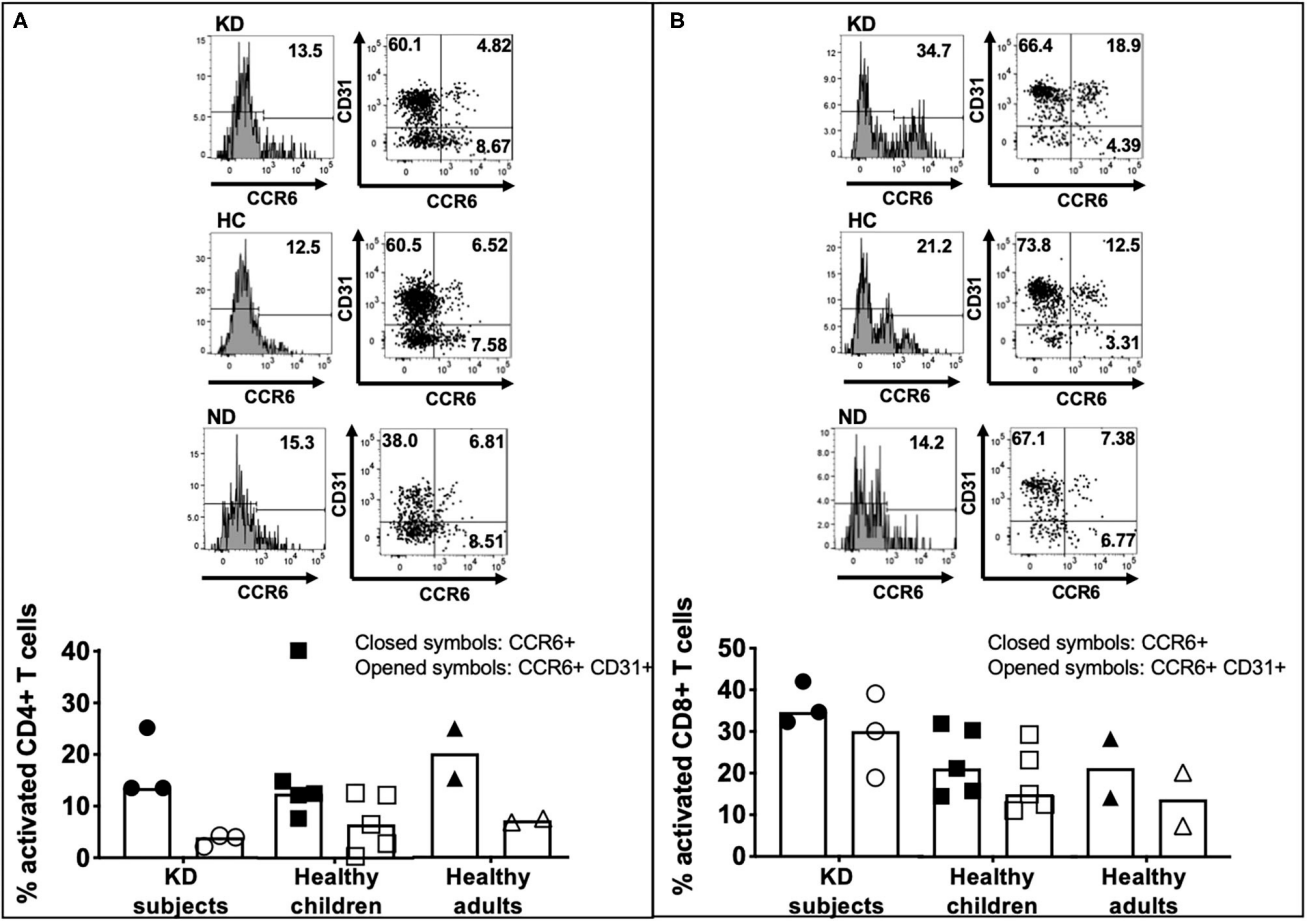
CCR6 and CD31 expression on activated LCWE-specific CD4+ and CD8+ T cells. To address the ability of LCWE-specific CD4+ and CD8+ T cells to home to the vessels (CCR6) and their potential role in the pathogenesis of vascular inflammation, the expression of CCR6 and CD31 on activated LCWE-specific T cells was studied. PBMC from subacute KD subjects (*n* = 3; KD 5–7, Table 1), healthy children (*n* = 5; HC 5–9, Table 1), and healthy adults (*n* = 2; ND3–4, Table 1) were stimulated with LCWE (1 μg/ml) for 4 days and the expression of CCR6 and CD31 on CD4+ HLA-DR+ **(A)** and CD8+ HLA-DR+ **(B)** T cells were studied by flow cytometry. A representative FACS plot of CCR6+ and CCR6+ CD31+ T cells from each cohort is shown. Activated CD4+ T cells showed a higher prevalence of CCR6+ CD31- (4.85–32.3%) than CCR6+ CD31+ (0.36–12.6%) suggesting that if these cells homed to the vessels, they could promote inflammation. Activated CD8+ T cells showed higher expression of CD31+ within the CCR6+ T cells than CD4+ T cells (7.38–39.1%) vs. CD31- (2.87–8.87%). The data are shown as scatter dot plots with median and interquartile range. Each symbol represents the results derived from each subject tested. Closed symbols: percent activated CD4+ or CD8+ T cells express CCR6. Opened symbols: percent activated CD4+ or CD8+ T cells express both CCR6 and CD31. The statistical differences between cohorts were tested by one-way ANOVA. A *P* < 0.05 was considered significant. No difference of CCR6+ and CCR6+ CD31+ expression on CD4+ (CCR6+, *p* = 0.9489; CCR6+ CD31+, *p* = 0.5132) and CD8+ (CCR6+, *p* = 0.2563; CCR6+ CD31+, *p* = 0.1678) T cells were found among the 3 cohorts.

Higher numbers of LCWE-specific CD8+ T cells expressed CCR6 compared to CD4+ T cells (Figure 5A) in subacute KD subjects (32.3–42.0% vs. 13.5–25.2%), healthy children (14.5–31.9% vs. 7.7–40.2%), and healthy adults (14.2–28.3 vs. 15.3–25.0) (Figure 5; Supplementary Figure 4). CCR6+ CD4+ T cells had lower expression of CD31 than CCR6+ CD8+ T cells: 2.17–4.28% in KD children; 0.36–12.6% in healthy children; 6.81–7.51% in healthy adults (Figure 5A; Supplementary Figure 4). Activated CD8+ T cells that co-expressed CCR6 and CD31 were numerous: 18.9–30.9% in KD children; 11.0–29.3% in healthy children; 7.38–20.2% in healthy adults (Figure 5B; Supplementary Figure 4). Taken together, the results suggest that activated LCWE-specific T cells have the potential to promote inflammation in the vascular compartment.

## Discussion

Here we addressed the T cell response to LCWE in KD subjects compared to healthy children and healthy adult donors. LCWE induces a vasculitis in mice following intraperitoneal injection, which is defined by the activation and infiltration of innate cells and CD8+ cytotoxic T cells into vascular tissues and the development of acute inflammation of the coronary arteries and the aorta. Mice develop aneurysms and myocarditis resembles the immunopathology and cardiac lesions observed in children with KD (4, 6, 13).

We recently reported that the immune phenotype of acute KD children suggests different antigenic exposures that together with genetic traits leads to coronary artery inflammation (14). This study demonstrated that LCWE-specific T cells are expand upon stimulation not only in subacute KD but also in healthy pediatric and adult individuals supporting the concept that LCWE is unlikely to be trigger for KD. However, the relevant Th17 and iTreg response to LCWE extract suggested that the uptake of the antigen by professional antigen presenting cells (APC) stimulated IL-1β signaling, which, along with IL-6, is indispensable for Th17 development (9). In mice, IL-1β signaling is the main pathogenic event that leads to vasculitis and myocarditis with both processes prevented by anakinra, an IL-1 receptor antagonist (5, 15).

LCWE extract-specific CD8+ T cells express CCR6, a homing receptor for the vessels and the heart (together with CD31) and the gut suggesting that, depending upon stimulatory and homing conditions, these cells could potentially harm the vascular compartment and affect the permeability of the gut, which is a feature of the murine model of KD (6, 7).

The study has both strengths and limitations. This is the first demonstration of an LCWE extract-specific T cell repertoire in subacute KD and healthy children. The lack of access to tissues leaves open the question of whether certain priming conditions could potentially lead to a pro-inflammatory T cell response that results in the vasculitis of KD.

In conclusion, both children and adults possess an LCWE-specific T cell repertoire that can be stimulated to express surface markers mediating homing to the vessels. However, there is currently no evidence that these cells actually participate in vascular inflammation in humans.

## Data Availability Statement

The original contributions presented in the study are included in the article/Supplementary Material, further inquiries can be directed to the corresponding author/s.

## Ethics Statement

The studies involving human participants were reviewed and approved by Institutional Review Board at the University of California, San Diego. Written informed consent to participate in this study was provided by the participants' legal guardian/next of kin.

## Author Contributions

L-EH in the Franco's laboratory executed the experiments, generated the figures, and participated to the manuscript preparation. MA and MN provided the *Lactobacillus casei* cell wall extract (LCWE) for the work. JB and AT enrolled Kawasaki disease patients and healthy children for the study. AF directed the work and wrote the manuscript. All authors contributed to the article and approved the submitted version.

## Conflict of Interest

The authors declare that the research was conducted in the absence of any commercial or financial relationships that could be construed as a potential conflict of interest.

## Supplementary Material

Supplementary Figure 1 Quality control for LCWE extract. LCWE has been tested by running in a PAGE gel with LPS as a control then stained with Pro-Q Emerald 300 polysaccharide staining kit. LPS like bands were absent in the LCWE extract preparation.

Supplementary Figure 2 Activation of CD4+ and CD8+ T in response to anti-CD3 and anti-CD28 agonistic antibodies. PBMC from the subjects studied for CD4+ and CD8+ T cell response to LCWE were also stimulated with 1 μg/ml of anti-CD3 and anti-CD28 agonistic antibodies for 4 days in the absence of exogenous IL-2. The percentages of activated CD4+ HLA-DR+ **(A)** and activated CD8+ HLA-DR+ **(B)** T cells were enumerated by flow cytometry.

Supplementary Figure 3 Effector and central memory LCWE-specific CD4+ and CD8+ T cells. IL-15R and CCR7 expression on CD4+ HLA-DR+ and CD8+ HLA-DR+ T cells from all the subjects tested (3 KD subjects, 5 healthy children, and 2 healthy adults). PBMC were stimulated with 1 μg/ml of LCWE for 4 days and CCR6 and CD31 expression were studied by flow cytometry.

Supplementary Figure 4 CCR6 and CD31 expression on activated LCWE-specific CD4+ and CD8+ T cells. CCR6 and CD31 expression on CD4+ HLA-DR+ and CD8+ HLA-DR+ T cells from all the subjects tested (3 KD subjects, 5 healthy children, and 2 healthy adults). PBMC were stimulated with 1 μg/ml of LCWE for 4 days and CCR6 and CD31 expression were studied by flow cytometry.
