# Supplementary figures and images for: Characterization of the T Cell Response to Lactobacillus casei Cell Wall Extract in Children With Kawasaki Disease and Its Potential Role in Vascular Inflammation
Source: Front Pediatr. 2021 Feb 19;9:633244. doi: 10.3389/fped.2021.633244 (PMC7933244; doi:10.3389/fped.2021.633244)

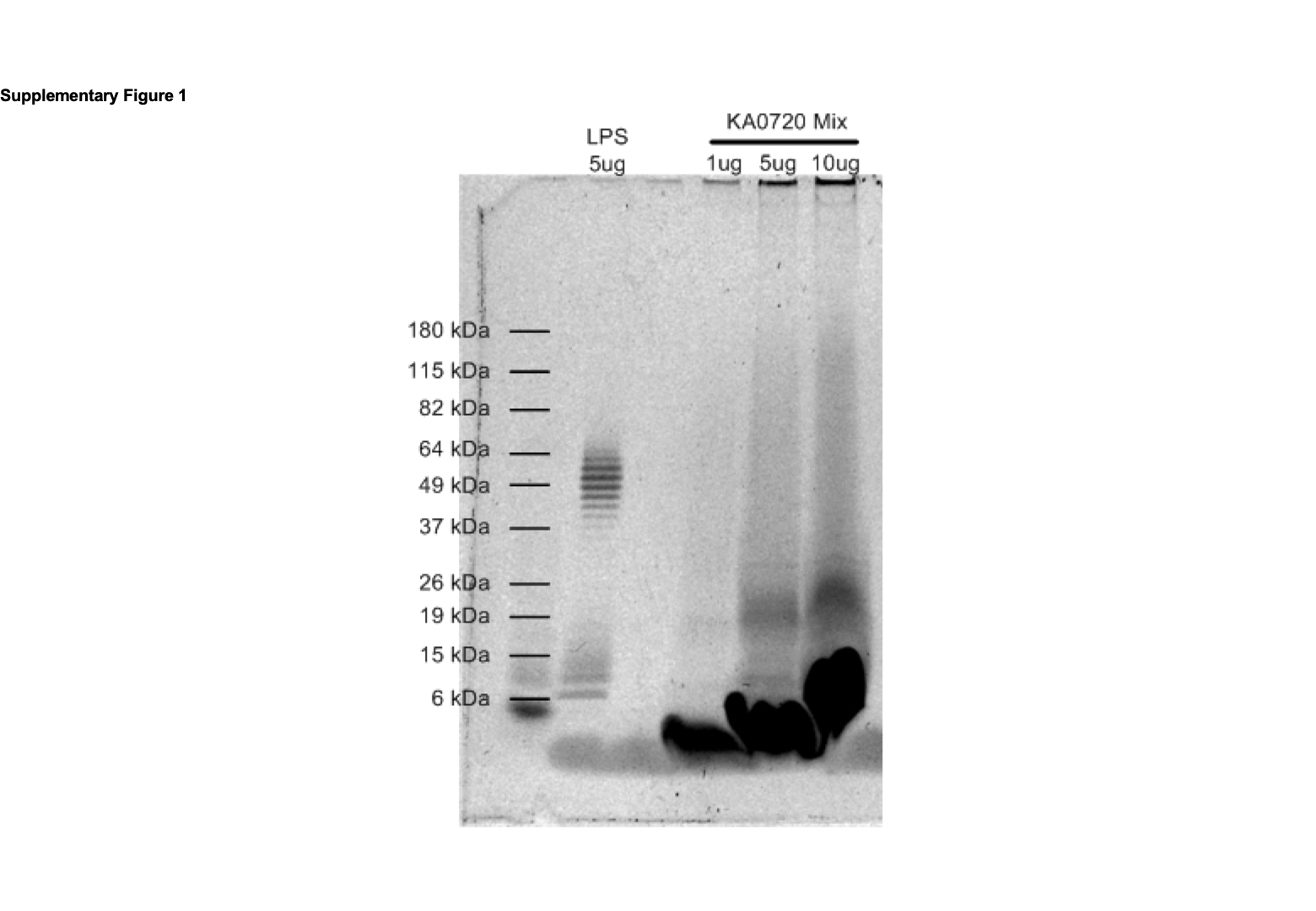

Supplement: Supplementary Figure 1 — Quality control for LCWE extract. LCWE has been tested by running in a PAGE gel with LPS as a control then stained with Pro-Q Emerald 300 polysaccharide staining kit. LPS like bands were absent in the LCWE extract preparation. [file Image_1.TIFF]
